# Non-native fold of the putative VPS39 zinc finger domain

**DOI:** 10.12688/wellcomeopenres.16078.2

**Published:** 2020-08-12

**Authors:** Benjamin G. Butt, Edward J. Scourfield, Stephen C. Graham

**Affiliations:** 1 Department of Pathology, University of Cambridge, Cambridge, CB2 1QP, UK

**Keywords:** CORVET, membrane trafficking, zinc finger domain, class C core

## Abstract

**Background:** The multi-subunit homotypic fusion and vacuole protein sorting (HOPS) membrane-tethering complex is involved in regulating the fusion of late endosomes and autophagosomes with lysosomes in eukaryotes. The C-terminal regions of several HOPS components have been shown to be required for correct complex assembly, including the C-terminal really interesting new gene (RING) zinc finger domains of HOPS components VPS18 and VPS41. We sought to structurally characterise the putative C-terminal zinc finger domain of VPS39, which we hypothesised may be important for binding of VPS39 to cellular partners or to other HOPS components.

**Methods: **We recombinantly expressed, purified and solved the crystal structure of the proposed zinc-binding region of VPS39.

**Results:** In the structure, this region forms an anti-parallel β-hairpin that is incorporated into a homotetrameric eight-stranded β-barrel. However, the fold is stabilised by coordination of zinc ions by residues from the purification tag and an intramolecular disulphide bond between two predicted zinc ligands.

**Conclusions: **We solved the structure of the VPS39 C-terminal domain adopting a non-native fold. Our work highlights the risk of non-native folds when purifying small zinc-containing domains with hexahistidine tags. However, the non-native structure we observe may have implications for rational protein design.

## Introduction

Eukaryotic cells use an interconnected system of membrane-bound compartments to partition intracellular space, allowing a multitude of biological reactions to proceed simultaneously in distinct chemical environments. The primary carriers of macromolecules between these compartments are vesicles, which bud from donor membranes in a cargo-dependent manner before fusing with an acceptor membrane at the destination compartment. Membrane fusion in the endomembrane system is critically dependent on SNARE (soluble *N*-ethylmaleimide sensitive factor attachment protein receptor) proteins, the co-folding of which on opposing membranes provides the energy for membrane bilayer mixing and thus vesicle fusion ^1^. SNARE activity is tightly regulated by both Sec1/Munc18 family proteins, which bind directly to SNAREs, and by multi-protein ‘tethering’ complexes that bring vesicles into close apposition to allow the physical contact of SNARE proteins on opposing membranes ^2^. The conserved multi-subunit tethering complexes CORVET (class C core vacuole/endosome tethering) and HOPS (homotypic fusion and vacuole protein sorting) combine both of these activities by incorporating the Sec1/Munc18 family protein VPS33A ^3– 5^. CORVET mediates homotypic fusion of early endosomes ^6^, while HOPS mediates heterotypic fusion of late endosomes with lysosomes ^4, 5^ and autophagosomes with lysosomes ^7– 9^.

The human CORVET and HOPS complexes share four conserved core subunits (VPS11, VPS16, VPS18, VPS33A), known collectively as the class C core ^3, 10^. Two additional, unique subunits direct each complex to its respective membrane target; VPS8 and TRAP1 direct CORVET to Rab5-positive membranes ^6, 11^, while VPS41 and VPS39 direct HOPS to Rab7-positive membranes ^12, 13^. Previous studies using truncation mapping have highlighted the importance of the C-terminal regions of HOPS components in assembly of the HOPS complex ^3, 14– 16^. Recruitment of VPS41 to the class C core is facilitated by the C-terminal RING (really interesting new gene) domains of VPS18 and VPS41, which interact directly ^15^. RING domains are a type of zinc finger, with an eight-residue motif containing six or seven cysteine residues and one or two histidine residues that coordinate two zinc ions ^17– 19^. RING domains may be involved in protein-protein, protein-lipid or protein-nucleic acid interactions, and have a wide variety of cellular functions ^17– 19^.

**Figure 1.**
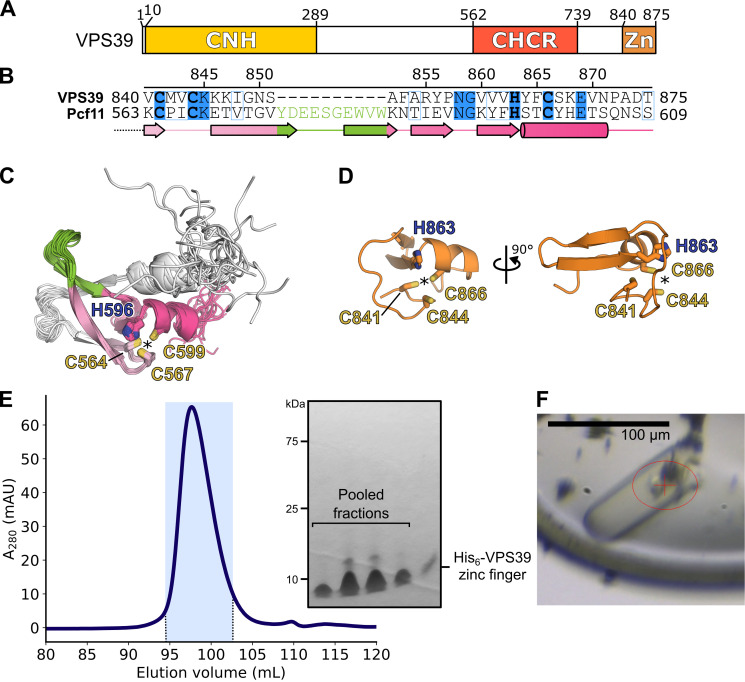
Purification and crystallisation of the VPS39 zinc finger domain. ( **A**) Predicted domain organisation of human VPS39, showing the position of the short C-terminal zinc finger domain that is the focus of this study (CNH, citron homology domain; CHCR, clathrin heavy chain repeat; Zn, zinc finger domain). Predicted domain boundary residue numbers are indicated above the schematic. ( **B**) Sequence alignment of the VPS39 C-terminal domain (top) and the conserved region of the Pcf11 zinc finger domain (bottom). Residues that are identical (blue shading) or share similar chemical properties (blue outlines) are indicated. Zinc ligands in Pcf11 and predicted zinc ligands in VPS39 are shown in **bold**. Residues numbers above the sequence are for VPS39, the secondary structure of Pcf11 is shown below and the extended β-hairpin region of Pcf11 that is not conserved in VPS39 is shown in green. ( **C**) Solution NMR structure of the Pcf11 zinc finger domain (PDB ID: 2NAX) ^20^ showing 20 lowest energy conformers in ribbon representation, with side chains involved in coordinating zinc ions shown as sticks. The region that is conserved between Pcf11 and VPS39 is coloured light pink to dark pink (N to C terminus), the extended β-hairpin being shown in green as in (B). The approximate position of the bound zinc ion in each conformer is shown by an asterisk (*). ( **D**) Homology model of VPS39 C-terminal domain based on S. cerevisiae Pcf11 zinc finger domain. Putative zinc ligands are shown as sticks and the approximate position of a bound zinc ion is indicated by an asterisk. ( **E**) SEC elution profile of purified VPS39 C-terminal domain (UV absorbance: solid blue line). Fractions that were analysed by SDS-PAGE (inset) are highlighted in light blue. (Inset). SDS-PAGE analysis of SEC elution fractions 35–39 (94.1–102.1 mL). SEC fractions pooled for crystallisation experiments are indicated and approximate positions of molecular weight markers are shown. ( **F**) Crystal of purified VPS39 C-terminal domain mounted on beamline I04 at the Diamond Light Source (scale bar = 100 μm; position of X-ray beam: red crosshair).

The C terminus of VPS39 contains a putative zinc finger domain ^15^ ( Figure 1A), the closest homologue of which is the zinc finger domain of *Saccharomyces cerevisiae* protein Pcf11 ( Figure 1B, C) ^20^. This putative VPS39 zinc finger domain is much shorter than those of VPS18 and VPS41, and is predicted to bind only one zinc ion via four ligands ^15^. Given that VPS41 is recruited to the class C core by an interaction between two zinc finger domains ^14^, and that the C-terminal region of VPS39 is required for its interaction with VPS11 ^16^, we hypothesised that the putative VPS39 C-terminal zinc finger domain may be required for its incorporation into the HOPS complex or for binding other cellular partners.

There is currently no high-resolution structural information available for any region of human VPS39, nor its yeast homologue vps39 (a.k.a. vam6). An atomic-resolution structure of the putative VPS39 zinc finger domain may further our understanding of HOPS complex assembly and function. We solved the structure of crystals formed by the VPS39 zinc finger domain to 2.9 Å resolution, but observed that the protein had adopted a non-native fold mediated by interactions between zinc ions and the purification tag.

## Methods

### Protein expression and purification

Residues 840–875 of human VPS39 isoform 2 (UniProt ID Q96JC1-2), corresponding to the putative C-terminal zinc finger domain, were cloned into pOPTH, (derived from pOPT ^21^), with an N-terminal MetHis _6_ purification tag and expressed in *Escherichia coli* strain BL21(DE3) pLysS. Bacteria were cultured in 2×TY medium, recombinant proteins being expressed overnight at 22°C following addition of 0.4 mM isopropyl β- d-1-thiogalactopyranoside. Cultures were harvested by centrifugation at 5000×g for 15 min and cell pellets were stored at -80°C.

Bacterial cell pellets were resuspended in lysis buffer (20 mM TRIS pH 7.5, 500 mM NaCl, 20 mM imidazole pH 7.5, 0.5 mM MgCl _2_, 1.4 mM β-mercaptoethanol, 0.05% Tween-20) supplemented with 400 U bovine pancreas DNase I (Merck) and 200 μL EDTA-free protease inhibitor cocktail (Merck) at 4°C. Cells were lysed using a TS series cell disruptor (Constant Systems) at 24 kPSI and the lysate was cleared by centrifugation at 40,000×g for 30 min at 4°C. The cleared lysate was incubated with Ni ^2+^-nitrilotriacetic acid agarose resin (Qiagen) equilibrated in wash buffer (20 mM TRIS pH 7.5, 500 mM NaCl, 20 mM imidazole pH 7.5) for 1 h at 4°C before being applied to a column and washed with >10 column volumes of wash buffer. Bound protein was eluted using elution buffer (20 mM TRIS pH 7.5, 500 mM NaCl, 250 mM imidazole pH 7.5), concentrated, and further purified by size-exclusion chromatography (SEC) using an S75 16/600 column (GE Healthcare) equilibrated in SEC buffer (20 mM TRIS pH 7.5, 200 mM NaCl, 1 mM dithiothreitol (DTT)). After storage overnight at 4°C, purified VPS39 was concentrated using 3 kDa nominal molecular weight cut-off centrifugal concentrators (Millipore) and subjected to crystallisation trials as described below. Protein concentrations were estimated from absorbance at 280 nm using a calculated extinction coefficient ^22^ for VPS39(840–875), assuming all cysteines were reduced.

### X-ray crystallography

VPS39(840–875) was crystallised in sitting drops by mixing 200 nL of 19.4 mg/mL protein in SEC buffer with 200 nL of reservoir solution (100 mM HEPES pH 7.5, 200 mM ammonium acetate, 45% (v/v) 2-methyl-2,4-pentanediol (MPD)) and equilibrating against 80 µL of reservoir at 20°C for 30 months. The VPS39 crystal was cryo-cooled by plunging into liquid nitrogen, no cryopreservant being added as the high concentration of MPD in the reservoir solution was predicted to provide sufficient cryoprotection. Diffraction data were recorded at 100 K on a Pilatus 6M-F detector (Dectris) at Diamond Light Source beamline I04. Data were collected in three sweeps, as shown in Table 1.

**Table 1.** Data collection strategy. Data were recorded from a single crystal in the order Peak 1, Peak 2 and then High-energy remote.

Dataset	Peak 1	Peak 2	High-energy remote
Wavelength (Å)	1.2810	1.2810	0.9795
Exposure (s)	0.5	0.2	0.2
X-ray transmission (%)	3.0	29.9	52.4
Oscillation per frame (°)	0.2	0.2	0.2
Total number of frames	900	900	1800

Images were processed using DIALS version 1.14.13 ^23^ then CCP4 suite version 7.0.078 ^24^ programs POINTLESS version 1.11.21 ^25^ and AIMLESS version 0.7.4 ^26^ as implemented by the xia2 version 0.5.902 data processing pipeline ^27^. Data collection statistics are shown in Table 2. Two-wavelength multiple anomalous dispersion analysis was performed using the CCP4 suite version 7.1.001 ^24^ CRANK2 version 2.0.229 automated experimental phasing pipeline ^28^, with substructure determination performed with SHELXD version 2019/1 ^29^, density modification performed with Parrot version 0.8 ^30^, and iterative model building and refinement performed with Buccaneer version 1.1 ^31, 32^ and Refmac5 version 5.8.0258 ^33^. Cycles of iterative manual building with COOT version 0.8.9 ^34^ and TLS plus positional refinement using Refmac5 version 5.8.0258 ^33^ with local non-crystallographic symmetry (NCS) restraints were initially performed using the high-energy remote wavelength dataset ( Table 2). Building was assisted by the use of real-time molecular dynamics-assisted model building and map fitting with ISOLDE version 1.0b3 ^35^. To ameliorate radiation damage evident in the structure, later stages of refinement were performed using the first 300 frames of the second peak wavelength dataset (Peak 2; Table 1), processed using xia2 as above with the same set of reflections kept ‘free’ for cross-validation ^36^. Final cycles of refinement were performed using autoBUSTER version 2.10.3 ^37^ with local NCS restraints and bond length/angle restraints for zinc ligands to ensure chemically-plausible zinc coordination ^38^. The quality of the model was monitored throughout refinement using MolProbity version 4.5.1 ^39^ and the validation tools in COOT version 0.8.9 ^34^. Refinement statistics are shown in Table 2. Molecular images were produced in PyMOL 2.4.0a0 Open-Source ^40^ and figures were composed in Inkscape version 1.0 ^41^. VPS39 C-terminal domain residues predicted to bind zinc were identified via generation of a homology model using I-TASSER version 5.1 ^42^ with the structure of *S. cerevisiae* Pcf11 (PDB ID: 2NAX) ^20^ as the template.

**Table 2.** Data collection and refinement statistics. The ‘Peak’ column describes the merged diffraction data from sweeps ‘Peak 1’ and ‘Peak 2’ ( Table 1) used for structure solution. The ‘Peak(1–300)’ column describes the subset of ‘Peak 2’ diffraction data used for structure refinement. Values in parentheses describe the high-resolution shell.

Dataset	Peak	High energy remote	Peak(1–300)
***Data collection***			
Wavelength (Å)	1.28096	0.97949	1.28096
Space group	*P* 4 _2_ 2 2	*P* 4 _2_ 2 2	*P* 4 _2_ 2 2
Cell dimensions			
*a, b, c* (Å)	104.17, 104.17, 39.43	104.17, 104.17, 39.43	104.18, 104.18, 39.42
*α, β, γ* (°)	90.0, 90.0, 90.0	90.0, 90.0, 90.0	90.0, 90.0, 90.0
Resolution (Å)	73.65–3.07 (3.12–3.07)	28.89–2.98 (3.03–2.98)	46.59–2.90 (2.95–2.90)
Total reflections	97,700 (2367)	116,580 (4961)	21,099 (1058)
Unique reflections	4399 (197)	4793 (247)	5148 (247)
Completeness (%)	100.0 (100.0)	100.0 (100.0)	99.3 (100.0)
Anomalous completeness (%)	100.0 (100.0)	99.9 (100.0)	96.8 (99.0)
Multiplicity	22.2 (12.0)	24.3 (25.7)	4.1 (4.3)
Anomalous multiplicity	12.6 (6.5)	13.7 (14.1)	2.3 (2.3)
*R _merge_*	0.121 (0.844)	0.124 (1.154)	0.075 (1.178)
*R _pim_*	0.027 (0.252)	0.026 (0.231)	0.041 (0.632)
CC _1/2_	0.998 (0.939)	0.999 (0.944)	0.966 (0.602)
CC _anom_	0.699 (0.011)	0.403 (0.020)	0.580 (-0.144)
Mean I/σ(I)	16.4 (2.4)	16.0 (3.1)	10.1 (0.9)
***Refinement***			
Resolution (Å)			46.59–2.90 (2.98–2.90)
Reflections			
Working set			4853 (350)
Test set			286 (18)
*R*_work_			0.2376 (0.2535)
*R*_free_			0.2686 (0.3028)
No. of atoms			
Protein			922
Solvent			1
Zinc ions			3
Root mean square deviation			
Bond lengths (Å)			0.008
Bond angles (°)			1.07
MolProbity score			2.05
Ramachandran favoured (%)			92.73
Ramachandran outliers (%)			0.00
Poor rotamers (%)			4.90
Mean B value (A ^2^)			122.04

## Results

The C-terminal region of human VPS39 contains a putative zinc finger domain (residues 840–875, Figure 1A) with four predicted zinc-binding residues (Cys841, Cys844, His863, Cys866). These residues are predicted to coordinate a single zinc ion based on homology to the zinc finger domain of *S. cerevisiae* protein Pcf11 ( Figure 1B–D). The coordinates for this theoretical model are available (see *Underlying data*) ^43^.

The VPS39 C-terminal domain was expressed with an N-terminal His _6_ tag in *E. coli* and purified using nickel affinity capture followed by SEC. The protein eluted from SEC as a single, symmetrical peak near the end of the elution profile ( Figure 1E), consistent with expectations for a small folded protein domain. Analysis of the eluted fractions by SDS-PAGE showed a single predominant band that migrated as would be expected for the VPS39 zinc finger domain (5.1 kDa; Figure 1E), with a much less intense band at higher apparent molecular mass that was presumed to be a small amount of SDS-resistant VPS39 dimer. The protein was concentrated and sparse matrix crystallisation screening was performed, but no crystals were obtained in the following six weeks. Approximately 30 months later, the crystallisation trays were re-inspected prior to disposal and a single crystal was observed ( Figure 1F). This crystal was harvested and diffraction data were recorded at two wavelengths ( Table 2), allowing the structure of the VPS39 zinc finger domain to be solved using anomalous dispersion signal from the incorporated zinc ions. The model was initially refined against the high-energy data, but later stages of the refinement proved challenging because map features were indistinct and loop density was poor. We were concerned that intense X-ray exposure during data collection at the peak wavelength, where the zinc ions would have a large X-ray absorption cross-section ^44^, may have caused radiation damage. The final stages of refinement were thus performed using data recorded in the first 300 frames of the second sweep at the peak wavelength ( Table 1 and Table 2), which represented the best compromise between total X-ray exposure/damage and data redundancy/resolution. The structure was refined to 2.90 Å resolution with residuals *R* = 0.238, *R*_free_ = 0.269 and good stereochemistry, with an overall MolProbity score ^39^ of 2.05 ( Table 2). The structure is available under PDB ID: 6ZE9; raw diffraction images, crystallographic datasets and X-ray fluorescence scans are available (see *Underlying data*) ^45^.

**Figure 2.**
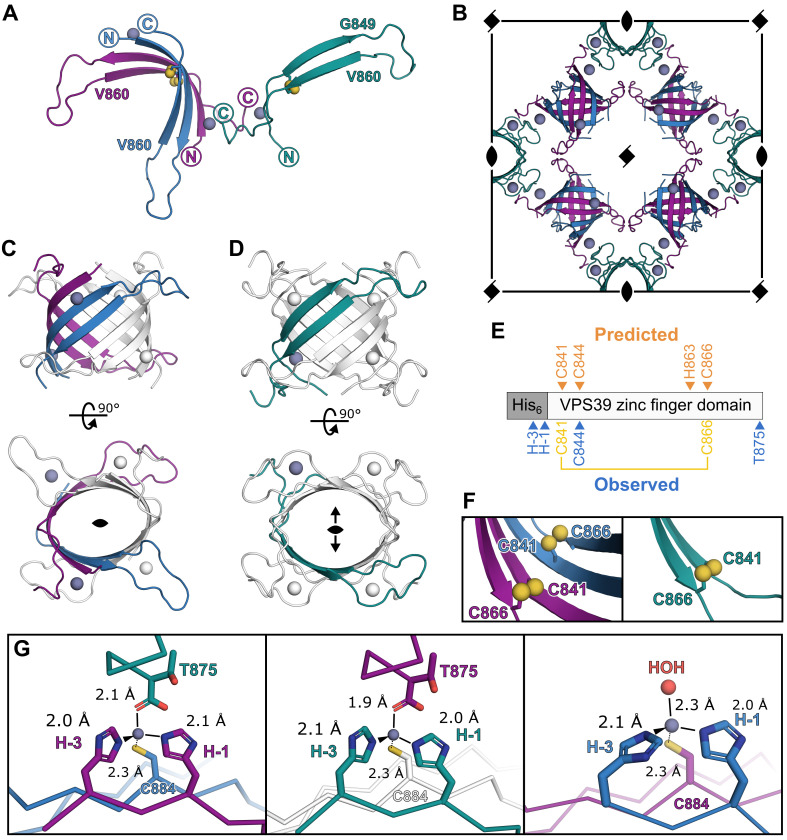
Non-native structure of VPS39 C-terminal domain. ( **A**) Three copies of the VPS39 C-terminal domain in the asymmetric unit, showing the antiparallel β-hairpin fold of each molecule. VPS39 is shown in ribbon representation with N and C termini shown. Cysteine sulphur atoms that form disulphide bonds are shown as yellow spheres and zinc ions are shown as grey spheres. Residues at the start and end of the loop that joins the two β-strands are indicated. ( **B**) Unit cell of the VPS39 crystal lattice viewed along the *c* axis, showing eight stranded β-barrels formed by symmetry-related VPS39 chains. Selected symmetry axes (four-fold screw and two-fold rotation) are indicated using standard symmetry symbols. Incorporated zinc ions are shown as spheres. ( **C**, **D**) Eight-stranded β-barrels formed by symmetry-related chains via a single crystallographic two-fold rotational symmetry axis ( **C**) or two orthogonal two-fold rotational axes ( **D**) are shown in ribbon representation. The unique VPS39 molecules from the asymmetric unit ( **A**) are coloured blue/purple ( **C**) or teal ( **D**), with symmetry-related chains shown in light grey. Zinc ions coordinated by visible residues are shown as spheres. Two orthogonal views are shown, with symmetry axes shown in the bottom view where arrows represent a two-fold rotational axis in the plane of the image. ( **E**) Schematic of the His _6_-VPS39 zinc finger construct used in this study. (Top) Residues predicted to coordinate zinc ions by homology to *S. cerevisiae* Pcf11 ( Figure 1B–D) are denoted with arrows. (Bottom) Residues that coordinate zinc ions in the crystal structure, including two residues from the purification tag, are denoted with arrows. Residues involved in the intramolecular disulphide bond in each chain are joined. ( **F**) Enlarged views of intramolecular Cys841-Cys866 disulphide bond in each molecule of the VPS39 C-terminal domain. ( **G**) Enlarged views of the three zinc ions in the asymmetric unit. VPS39 backbone atoms are shown as lines, with side chains involved in coordinating zinc ions shown as sticks. Bond lengths between zinc ions and relevant side chain atoms or water molecules are indicated.

The asymmetric unit contains three copies of the VPS39 C-terminal domain: two full-length copies (residues 840–875; purple and teal in Figure 2A) and a third copy spanning residues 840–869 (blue in Figure 2A). The remaining C-terminal residues of the third copy are absent from the electron density and presumably disordered. Each copy of the VPS39 C-terminal domain forms an antiparallel β-hairpin, with residues 849–860 forming a loop linking the two β-strands ( Figure 2A). Strikingly, the VPS39 C-terminal domains are all organised around crystallographic symmetry axes such that they form eight-stranded β-barrels ( Figure 2B). There are two distinct homotetramers formed: the first comprises two NCS-related chains that interact with two additional chains that are related by crystallographic two-fold rotational symmetry ( Figure 2C), while the second homotetramer is formed by a single VPS39 C-terminal domain interacting with three additional chains that are related by two orthogonal two-fold crystallographic symmetry axes ( Figure 2D).

The asymmetric unit contains three zinc ions, consistent with the four predicted zinc ligands in each VPS39 copy based on homology to Pcf11 ( Figure 1B–D). All zinc ions have tetrahedral geometry. However, only one of the predicted zinc ligands (Cys844) is involved in zinc ion coordination ( Figure 2E). Of the remaining predicted zinc ligands, Cys841 and Cys866 had become oxidised to form an intramolecular disulphide bond in each VPS39 molecule ( Figure 2F) and the final predicted ligand (His863) is not in close proximity to the zinc ions. Instead, the remaining zinc ligands are provided by two histidine side chains from the MetHis _6_ purification tag (His-3 and His-1) and the terminal carboxylate group of the polypeptide chain (Thr875) or a water molecule ( Figure 2G). As two of the ligands for each zinc ion derive from the affinity purification tag and the fold of the VPS39 C-terminal domain that we observe differs significantly from that of the closest sequence homologue (compare Figure 1D and Figure 2A), we conclude that the observed fold is non-native.

## Discussion

We present the crystal structure of the human VPS39 zinc finger domain in a non-native fold. In the structure, three copies of the VPS39 C-terminal domain in the asymmetric unit ( Figure 2A) combine with symmetry-related chains to form two similar, homotetrameric, eight-stranded β-barrels ( Figure 2C, D). In each copy of VPS39, two of the residues predicted to bind zinc ions (Cys844 and Cys866; Figure 2E) instead form intramolecular disulphide bonds ( Figure 2F), with the remaining zinc ligands provided by side chains from the N-terminal His _6_ purification tag and the carboxylate group of the polypeptide chain or a water molecule ( Figure 2G).

Structural characterisation of VPS39 was undertaken to complement a yeast two-hybrid screen of HOPS component zinc finger domains, including the putative VPS39 zinc finger domain, with the aim of identifying cellular binding proteins ^15^. However, as pull-down experiments failed to validate any of the potential interactions that were tested, structural characterisation of the VPS39 C-terminal domain was not actively pursued. After 30 months, as the crystallisation trials were being discarded, a single VPS39 C-terminal domain crystal was identified and used for successful structure determination. It seems very likely that the non-native fold that we observed arose from re-folding of the purified VPS39 C-terminal domain during the extended crystallisation experiment. The elution of freshly purified VPS39 C-terminal domain from SEC ( Figure 1E) was consistent with this small protein being monomeric, whereas the β-barrels of VPS39 in the crystal structure would be likely to elute much earlier, although we concede that formation of a β-barrel fold from the outset remains possible and that the higher molecular mass band observed in SDS-PAGE may represent SDS-resistant β-barrels or other aberrantly folded forms of the VPS39 C-terminal domain.

Refolding of the VPS39 C-terminal domain to form the observed β-barrels is likely to have been promoted via the concerted actions of zinc binding by the purification tag, disulphide bond formation and formation of β-sheets with unsatisfied backbone hydrogen bonds. The histidine side chains from the MetHis _6_ purification tag could have competed with Cys841 and Cys866 for coordination of the zinc ions, thereby liberating the side chains of these two cysteine residues. While the VPS39 C-terminal domain was purified under reducing conditions (the SEC buffer being supplemented with 1 mM DTT), it is likely that the contents of the crystallisation drops became oxidised during their extended incubation. The liberated cysteine side chains may thus have formed the observed intramolecular disulphide bond, prohibiting them from competing with the MetHis _6_ tag side chains for re-binding to the zinc ion. Either or both molecular rearrangements could have promoted re-folding of the protein backbone to adopt the extended β-hairpin fold observed in this structure. The refolded VPS39 β-sheets would have unsatisfied backbone hydrogen bonds, which could have promoted similar refolding of additional VPS39 molecules (akin to nucleation of amyloid fibrils). Such stimulated refolding could promote further exchange of zinc ligands and disulphide bond formation, acting as a ratchet to increase the pool of refolded VPS39 for crystallisation. The covalent interaction between β-barrels, mediated by the carboxy terminus of the polypeptide binding to the zinc ions, would have promoted stability of the crystal once nucleated.

While the structure presented here does not provide biological insight into the organisation or function of the putative VPS39 C-terminal zinc finger domain, there are still useful lessons to be learned. Firstly, nickel-affinity chromatography should be used with caution when purifying zinc-binding proteins as the similar chemical properties of zinc and nickel can lead to competition between purification tag residues and native zinc ligands for zinc ions. If this purification strategy is used, constructs should be engineered to include a protease cleavage site that can be used to remove the purification tag before downstream applications, particularly those involving long incubations such as crystallisation. We have previously reported structures where purification tag residues give rise to folding artefacts ^46^ and where metal ions help mediate non-natural ‘swapped’ β-strand topologies of crystallised molecules ^47^. While His _6_ tags are generally benign for crystallisation and may indeed be beneficial in some cases ^48^, caution should be exercised when using them to purify small zinc-containing domains.

The non-native β-barrel fold of the VPS39 C-terminal domain we observe here highlights the power of metal ion coordination to strongly promote the stable (re)folding of proteins ^49^, especially given the simple sequence requirements for efficient zinc binding (cysteine and histidine side chains or carboxylate groups). As a result, it is not uncommon for such features to arise spontaneously ^50, 51^, as has been previously noted in studies on directed protein evolution. Small zinc finger domains are often highly thermostable and tolerant to sequence changes outside of the zinc ligands ^52^, which has led to their use as scaffolds for modular protein design ^53– 55^. Novel, non-native, metal ion-coordinating folds such as the VPS39 fold reported in this work are potentially less likely to interact with off-target cellular components when used as biologics ^56^. The non-native fold of the VPS39 C-terminal domain presented here therefore expands the number of protein scaffolds available for rational therapeutic design.

## Data availability

### Underlying data

Protein Data Bank: Non-native fold of the putative VPS39 zinc finger domain. Accession number 6ZE9; https://identifiers.org/rcsb/pdb:6ZE9.

Apollo: Crystallographic diffraction data for structure of the VPS39 C-terminal domain. https://doi.org/10.17863/CAM.53867 ^45^.

This project contains raw diffraction images, crystallographic datasets and X-ray fluorescence scans.

Apollo: Theoretical model of the VPS39 zinc finger domain. https://doi.org/10.17863/CAM.54503 ^43^.

This project contains atomic coordinates for the theoretical model of the VPS39 zinc finger domain shown in Figure 1D.

Data hosted with Apollo are available under the terms of the Creative Commons Attribution 4.0 International license (CC-BY 4.0).
