# Supplementary material for: Non-native fold of the putative VPS39 zinc finger domain
Source: Wellcome Open Res. 2020 Aug 12;5:154. Originally published 2020 Jul 1. [Version 2] doi: 10.12688/wellcomeopenres.16078.2 (PMC7384125; doi:10.12688/wellcomeopenres.16078.2)
Supplement: Peer review history

# Peer review: Reviewer response for version 2

**DOI:** 10.21956/wellcomeopenres.17811.r39932

**Published:** 2020-08-17

**Authors:** Daniel Kümmel

**Affiliations:** 1 Institute of Biochemistry, University of Münster, Münster, Germany

I have no further comments.

Is the work clearly and accurately presented and does it cite the current literature?

Yes

If applicable, is the statistical analysis and its interpretation appropriate?

Not applicable

Are all the source data underlying the results available to ensure full reproducibility?

Yes

Is the study design appropriate and is the work technically sound?

Yes

Are the conclusions drawn adequately supported by the results?

Partly

Are sufficient details of methods and analysis provided to allow replication by others?

Yes

Reviewer Expertise:

structural biology, membrane trafficking

I confirm that I have read this submission and believe that I have an appropriate level of expertise to confirm that it is of an acceptable scientific standard.

# Peer review: Reviewer response for version 1

**DOI:** 10.21956/wellcomeopenres.17642.r39468

**Published:** 2020-07-23

**Authors:** Lauren Jackson, Boyang Xie

**Affiliations:** 1 Department of Biological Sciences, Vanderbilt University, Nashville, TN, 37235, USA; 2 Department of Biological Sciences, Vanderbilt University, Nashville, TN, USA

This article reports a non-native X-ray crystallography structure of the small C-terminal zinc finger domain of VPS39, which is hypothesized to bind HOPS tethering complex subunits or other cellular partners. The observed structure adopts an antiparallel b-hairpin structure that in turn forms eight-stranded b-barrels in the crystal. The authors anticipated four residues (Cys841, Cys844, His863, Cys866) would coordinate the zinc ion, but instead the zinc ion is coordinated by non-canonical residues, including two histidines from the His _6_ affinity tag. The structure is further stabilized by an intramolecular disulfide bond formed between Cys841 and Cys866. The authors think refolding of the VPS39 domain happened during crystallization; they suggest Cys residues oxidized over an extended 30-month incubation period, which prevented them from competing with the MetHis _6 _to bind zinc ion. The authors propose His _6_ affinity tags should be used with caution in zinc-binding proteins, and suggest that non-native folds may be promising scaffolds in therapeutic protein design. This study is well-documented and well-presented. We suggest clarification regarding a few minor points in the final version.

Figure 1

1. We suggest the authors include a sequence alignment between Pcf11 and VPS39 zinc finger domains with key Zn-binding residues marked, since authors use Pcf11 as an expected model for VPS39. This would help the reader follow their logic with a clear visual representation of Cys/His residues predicted to bind zinc.
2. In Figure 1C, the higher bands were presumed to be an SDS-resistant VPS39 dimer. It’s possible both native and non-native folds already existed at that point. Were all fractions used for crystallization trials, or did the authors use only fractions containing the single band?
3. Which column was used in Figure 1C? Are standards available to support VPS39 molecular mass? 

Methods

1. The authors mentioned snap-freezing purified VPS39 for storage, but did not specify whether fresh or frozen protein was used in crystallization trials. Could the freeze-thaw cycle affect the protein fold? The authors might comment on whether fresh or frozen protein was used for crystallization set up.
2. Did authors include any zinc in purification buffers? Is this necessary for these proteins, or do the protein just acquire from solution during expression? 

Is the work clearly and accurately presented and does it cite the current literature?

Yes

If applicable, is the statistical analysis and its interpretation appropriate?

Yes

Are all the source data underlying the results available to ensure full reproducibility?

Yes

Is the study design appropriate and is the work technically sound?

Yes

Are the conclusions drawn adequately supported by the results?

Yes

Are sufficient details of methods and analysis provided to allow replication by others?

Yes

Reviewer Expertise:

Structural biology, biochemistry, biophysics, membrane trafficking

We confirm that we have read this submission and believe that we have an appropriate level of expertise to confirm that it is of an acceptable scientific standard.

# Response

**Published:** 2020-07-31

**Authors:** Benjamin G. Butt

**Affiliations:** Department of Pathology, University of Cambridge, UK

**This article reports a non-native X-ray crystallography structure of the small C-terminal zinc finger domain of VPS39, which is hypothesized to bind HOPS tethering complex subunits or other cellular partners. The observed structure adopts an antiparallel b-hairpin structure that in turn forms eight-stranded b-barrels in the crystal. The authors anticipated four residues (Cys841, Cys844, His863, Cys866) would coordinate the zinc ion, but instead the zinc ion is coordinated by non-canonical residues, including two histidines from the His6 affinity tag. The structure is further stabilized by an intramolecular disulfide bond formed between Cys841 and Cys866. The authors think refolding of the VPS39 domain happened during crystallization; they suggest Cys residues oxidized over an extended 30-month incubation period, which prevented them from competing with the MetHis6 to bind zinc ion. The authors propose His6 affinity tags should be used with caution in zinc-binding proteins, and suggest that non-native folds may be promising scaffolds in therapeutic protein design. This study is well-documented and well-presented. We suggest clarification regarding a few minor points in the final version.**

**Figure 1**

**We suggest the authors include a sequence alignment between Pcf11 and VPS39 zinc finger domains with key Zn-binding residues marked, since authors use Pcf11 as an expected model for VPS39. This would help the reader follow their logic with a clear visual representation of Cys/His residues predicted to bind zinc.**

**We thank the reviewer for this helpful suggestion. We have included the relevant alignment as Figure 1B.**

 In Figure 1C, the higher bands were presumed to be an SDS-resistant VPS39 dimer. It’s possible both native and non-native folds already existed at that point. Were all fractions used for crystallization trials, or did the authors use only fractions containing the single band?

 We have marked the fractions that were pooled, concentrated and used for crystallisation trials on the inset of Figure 1E. We agree that we can’t exclude the possibility that the non-native fold was present in the initial purified sample, and that the higher molecular weight band corresponded to an aberrantly folded protein. We have expanded the final sentence of the second paragraph of the discussion to explicitly mention this possibility.

**Which column was used in Figure 1C? Are standards available to support VPS39 molecular mass?**

 We confirm that the chromatogram in Figure 1E is from a preparative Superdex 75 16/600 size-exclusion column. We did not calibrate this column using molecular mass standards when performing the purification. We note that the VPS39 C-terminal domain peak eluted between 94 and 102 mL while the buffer components eluted at approximately 110 mL (small peak evident in Figure 1E), consistent with a small folded domain. However, as stated in the discussion, we can’t discount the possibility that at least some of the protein formed higher-order oligomers when purified.

**Methods**

**The authors mentioned snap-freezing purified VPS39 for storage, but did not specify whether fresh or frozen protein was used in crystallization trials. Could the freeze-thaw cycle affect the protein fold? The authors might comment on whether fresh or frozen protein was used for crystallization set up.**

 The protein used for crystallisation was freshly purified: following SEC purification the sample was stored overnight at 4°C, and the protein was concentrated and used for crystallisation the following day without being snap-frozen. We apologise for this ambiguity. We have updated the penultimate sentence of the second paragraph of the methods section to explicitly state how the sample used for crystallisation was handled, as follows:

*“After storage overnight at 4°C, purified VPS39 was concentrated using 3 kDa nominal molecular weight cut-off centrifugal concentrators (Millipore) and subjected to crystallisation trials as described below.”*

**Did authors include any zinc in purification buffers? Is this necessary for these proteins, or do the protein just acquire from solution during expression?**

 We did not supplement the growth medium purification buffers with zinc. Our experience is that zinc is effectively scavenged from bacterial expression medium [Graham et al. (2005) Biochemistry 44:13820–36] and that, once captured, zinc is retained by zinc finger proteins unless they are subjected to treatment with chelating agents. However, we note that others have observed increased solubility of zinc-containing proteins when they supplement the growth medium with exogenous zinc [Gillet et al. (2013) Journal of Molecular Biology 425:2423–35].

# Peer review: Reviewer response for version 1

**DOI:** 10.21956/wellcomeopenres.17642.r39359

**Published:** 2020-07-07

**Authors:** Daniel Kümmel

**Affiliations:** 1 Institute of Biochemistry, University of Münster, Münster, Germany

This article reports an interesting finding about the crystallization of a small domain in a non-native fold. In their efforts to understand the architecture of the HOPS tethering complex, the authors attempted the crystallization of a predicted zinc-binding RING finger domain of the complex subunit Vps39. The determined structure does not show a RING finger fold, but reveals that the protein adopts a beta-hairpin structure instead and forms a tetrameric beta-barrel structure in the crystal. This conformation is stabilized by a zinc ion, which does not interact with the expected native coordinating residues but non-canonical residues, including two histidines form the affinity tag used for purification. The structure is further stabilized by a disulfide bridge. The authors conclude that aging of the protein during a long (30 month) crystallization experiment and the presence of a Met-6xHis affinity tag led to refolding of the protein into a non-native structure in the crystallization set-up. They propose that the accidentally created beta-barrel might be a useful template for future protein engineering applications.

 The results of the study are well documented and the conclusions are clearly presented. I suggest including one additional aspect in the manuscript, which is to discuss if the recombinant protein might not have been properly folded from the beginning. The following points should be considered:

- SDS-PAGE analysis of SEC fractions showed the presence of an SDS-resistant species of Vps39 CTD during purification. This might arise from the beta-structures observed in the crystal already being present at this point.
- The used construct contains only 35 residues, which is extremely small. Although it contains all predicted zinc-coordinating sites, it might not comprise the full, stable domain.
- A structure-based sequence alignment of the predicted structure of Vps39 CTD (Fig 1B), the observed structure and the structure of Pcf11 zinc finger would be interesting in this context.

Minor point: the spelling of TRIS should be corrected

Is the work clearly and accurately presented and does it cite the current literature?

Yes

If applicable, is the statistical analysis and its interpretation appropriate?

Not applicable

Are all the source data underlying the results available to ensure full reproducibility?

Yes

Is the study design appropriate and is the work technically sound?

Yes

Are the conclusions drawn adequately supported by the results?

Partly

Are sufficient details of methods and analysis provided to allow replication by others?

Yes

Reviewer Expertise:

structural biology, membrane trafficking

I confirm that I have read this submission and believe that I have an appropriate level of expertise to confirm that it is of an acceptable scientific standard.

# Response

**Published:** 2020-07-31

**Authors:** Benjamin G. Butt

**Affiliations:** Department of Pathology, University of Cambridge, UK

**This article reports an interesting finding about the crystallization of a small domain in a non-native fold. In their efforts to understand the architecture of the HOPS tethering complex, the authors attempted the crystallization of a predicted zinc-binding RING finger domain of the complex subunit Vps39. The determined structure does not show a RING finger fold, but reveals that the protein adopts a beta-hairpin structure instead and forms a tetrameric beta-barrel structure in the crystal. This conformation is stabilized by a zinc ion, which does not interact with the expected native coordinating residues but non-canonical residues, including two histidines form the affinity tag used for purification. The structure is further stabilized by a disulfide bridge. The authors conclude that aging of the protein during a long (30 month) crystallization experiment and the presence of a Met-6xHis affinity tag led to refolding of the protein into a non-native structure in the crystallization set-up. They propose that the accidentally created beta-barrel might be a useful template for future protein engineering applications.**

**The results of the study are well documented and the conclusions are clearly presented. I suggest including one additional aspect in the manuscript, which is to discuss if the recombinant protein might not have been properly folded from the beginning. The following points should be considered:**

**SDS-PAGE analysis of SEC fractions showed the presence of an SDS-resistant species of Vps39 CTD during purification. This might arise from the beta-structures observed in the crystal already being present at this point.**

 We agree – we cannot unambiguously assert that all the protein adopted the native conformation at the time of initial purification. We have updated the final sentence of the second paragraph of the discussion to more clearly state this point:

*“The elution of freshly purified VPS39 C-terminal domain from SEC (Figure 1C) was consistent with this small protein being monomeric, whereas the tetrameric β-barrels of VPS39 in the crystal structure would be likely to elute much earlier, although we concede that formation of a β-barrel fold from the outset remains possible and that the higher molecular mass band observed in SDS-PAGE may represent SDS-resistant β-barrels or other aberrantly folded forms of the VPS39 C-terminal domain.”*

**The used construct contains only 35 residues, which is extremely small. Although it contains all predicted zinc-coordinating sites, it might not comprise the full, stable domain.**

 We agree that this predicted domain is small, but its small size is not without precedent. Several independently-folded C2H2 and CCHC zinc finger domains are less than 30 residues in length (see for example [Lee et al. (1989) Science 245:635–7] and [D’Souza and Summers (2004) Nature 586–90]). While we can’t unambiguously assert that our choice of domain boundary was correct given the non-native fold we observed, we believe the size of our construct is consistent with expectations for an isolated zinc-binding domain.

**A structure-based sequence alignment of the predicted structure of Vps39 CTD (Fig 1B), the observed structure and the structure of Pcf11 zinc finger would be interesting in this context.**

 We thank the reviewer for suggesting that we include a sequence alignment of the Pcf11 and VPS39 zinc-binding domains, which we have included as Figure 1B. We have also included a figure of the Pcf11 zinc finger domain, highlighting the region that is not conserved between Pcf11 and VPS39 Figure 1C. Given the divergence in the predicted versus observed folds of the VPS39 C-terminal domain, we fear that a structure-based sequence alignment would be difficult and potentially uninformative. We have thus not included a second sequence for the VPS39 C-terminal domain, based on the non-native β-barrel fold, in this alignment, but note that a comparison of zinc ligands is presented in Figure 2E.

**Minor point: the spelling of TRIS should be corrected**

 We thank the reviewer for pointing out the correct IUPAC abbreviation for TRIS. We have changed it throughout the manuscript.
